# Supplementary material for: Conservation of Imprinting and Methylation of MKRN3, MAGEL2 and NDN Genes in Cattle
Source: Animals (Basel). 2021 Jul 2;11(7):1985. doi: 10.3390/ani11071985 (PMC8300276; doi:10.3390/ani11071985)
Supplement: Supplementary file 1 [file animals-11-01985-s001.zip › animals-1183251-supplementary.pdf]

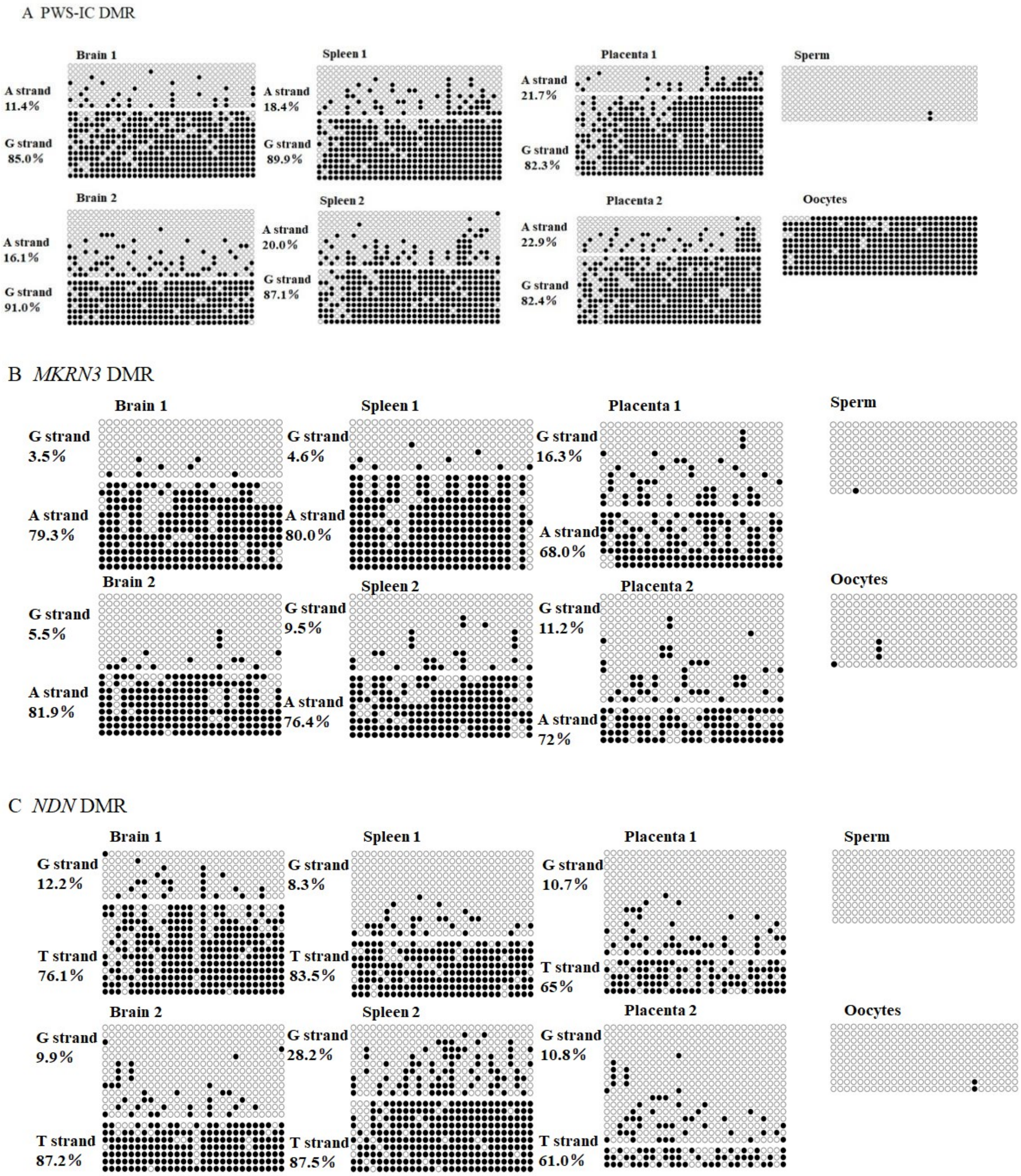


**Figure S1.** Methylation profiles of PWS-IC, *MKRN3* DMR and *NDN* DMR in bovine brain, spleen, placenta, sperms and oocytes. A: Methylation profiles of PWS-IC DMR. B: Methylation profiles of *MKRN3* DMR. **C.** Methylation profiles of *NDN* DMR . Methylated and unmethylated CpG dinucleotides are represented by filled and open circles, respectively.
